# Supplementary material for: Observation of structural switch in nascent SAM-VI riboswitch during transcription at single-nucleotide and single-molecule resolution
Source: Nat Commun. 2023 Apr 22;14:2320. doi: 10.1038/s41467-023-38042-2 (PMC10122661; doi:10.1038/s41467-023-38042-2)
Supplement: Supplementary file 1 — Supplementary Information [file 41467_2023_38042_MOESM1_ESM.pdf]

Supplementary Information

Observation of structural switch in nascent SAM-VI riboswitch during transcription at single-nucleotide and single-molecule resolution

Yanyan Xue[1,§], Jun Li[2,§], Dian Chen[1], Xizhu Zhao[3], Liang Hong[2,4,5]*, Yu Liu[1,5]*

[1] State Key Laboratory of Microbial Metabolism, School of Life Sciences and Biotechnology, Shanghai Jiao Tong University, Shanghai 200240, China. [2] School of Physics and Astronomy, Shanghai Jiao Tong University, Shanghai 200240, China. [3] Zhiyuan College, Shanghai Jiao Tong University, Shanghai 200240, China. [4] Institute of Natural Sciences, Shanghai Jiao Tong University, Shanghai 200240, China. [5] Shanghai Artificial Intelligence Laboratory, Shanghai 200232, China.

[§]The authors contribute equally.

Corresponding author: hongl3liang@sjtu.edu.cn; liuyu_sjtu@sjtu.edu.cn.

Contents

**Supplementary Figure 1**	**4**
**Supplementary Figure 2**	**5**
**Supplementary Figure 3**	**6**
**Supplementary Figure 4**	**7**
**Supplementary Figure 5**	**8**
**Supplementary Figure 6**	**9**
**Supplementary Figure 7**	**10**
**Supplementary Figure 8**	**11**
**Supplementary Figure 9**	**12**
**Supplementary Figure 10**	**13**
**Supplementary Figure 11**	**14**
**Supplementary Figure 12**	**15**
**Supplementary Figure 13**	**16**
**Supplementary Figure 14**	**17**
**Supplementary Figure 15**	**18**
**Supplementary Figure 16**	**19**
**Supplementary Figure 17**	**20**
**Supplementary Figure 18**	**21**
**Supplementary Figure 19**	**22**
**Supplementary Figure 20**	**23**
**Supplementary Figure 21**	**24**
**Supplementary Figure 22**	**25**
**Supplementary Figure 23**	**26**
**Supplementary Figure 24**	**27**
**Supplementary Figure 25**	**28**
**Supplementary Figure 26**	**29**
**Supplementary Figure 27**	**30**

**Supplementary Figure 28**	**31**
**Supplementary Figure 29**	**32**
**Supplementary Figure 30**	**33**
**Supplementary Figure 31**	**34**
**Supplementary Table 1**	**35**
**Supplementary Table 2**	**36**
**Supplementary Table 3**	**37**
**Supplementary Table 4**	**38**
**Supplementary Table 5**	**39**
**Supplementary Table 6**	**40**
**Supplementary Table 7**	**41**
**Supplementary Table 8**	**42**
**Supplementary Table 9**	**43**
**Supplementary Table 10**	**44**
**Supplementary Table 11**	**45**
**Supplementary Table 12**	**46**
**Supplementary Table 13**	**47**
**Supplementary Table 14**	**48**
**Supplementary Table 15**	**49**
**Supplementary Table 16**	**50**

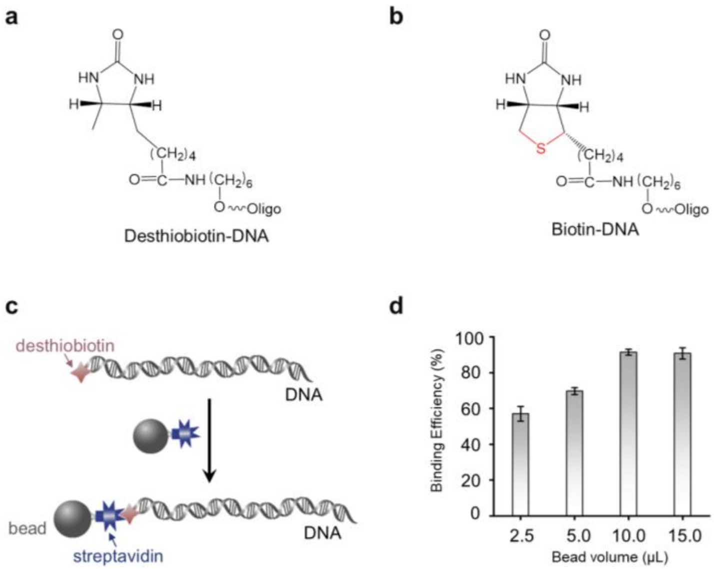

Supplementary Figure 1 │ Optimization of DNA-bead binding. The chemical structures of desthiobiotin (**a**) and biotin (**b**). **c,** Schematic diagram of desthiobiotin-DNA immobilization on streptavidin-beads. **d,** 1 nmole desthiobiotin-DNA incubated with 2.5, 5.0, 10.0 and 15.0 μL streptavidin-beads for 3 h, yielding the binding efficiencies higher than 90% for 10.0 or 15.0 μL beads. The data are presented as mean values ± s.d. for three independent experiments.

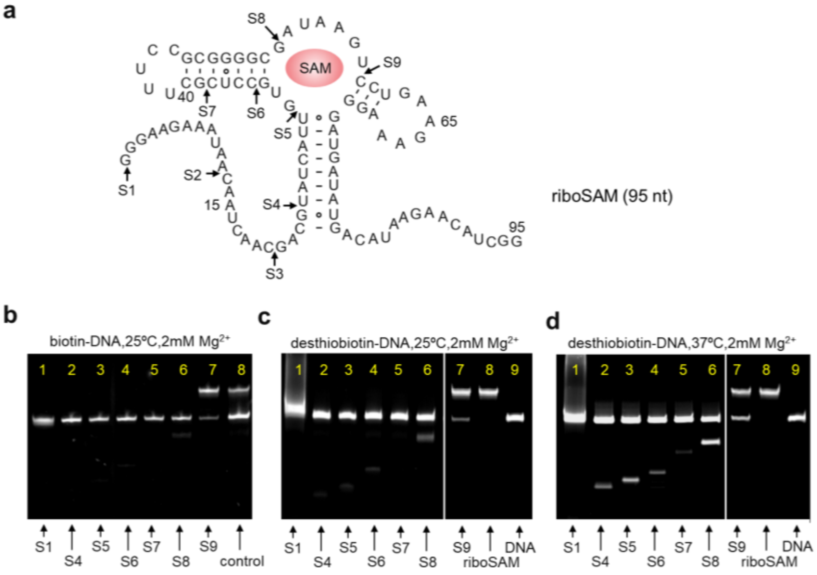

Supplementary Figure 2 │ Optimizing dissociation of the ECs from beads at different conditions. a, The secondary structure of riboSAM. 9-step hybrid-phase transcription was performed at different DNA templates and temperatures to optimize dissociation of ECs. The restart sites at each step were marked by arrows. **b-d,** The images of 12% denaturing PAGE of the liquid phase separated by SPE at steps 1 (Lane 1), 4 (Lane 2), 5 (Lane 3), 6 (Lane 4), 7 (Lane 5), 8 (Lane 6), 9 (Lane 7) using biotin-DNA templates at 25 ℃ (**b**), desthiobiotin-DNA templates at 25 ℃ (**c**), and desthiobiotin-DNA templates at 37 ℃ (**d**). Lane 8 at (**b**) contained the mixture of riboSAM and DNA template as control. Lanes 8 and 9 at (**c**) and (**d**) contained riboSAM and DNA template as control, respectively. The experiments were repeated independently for at least three times.

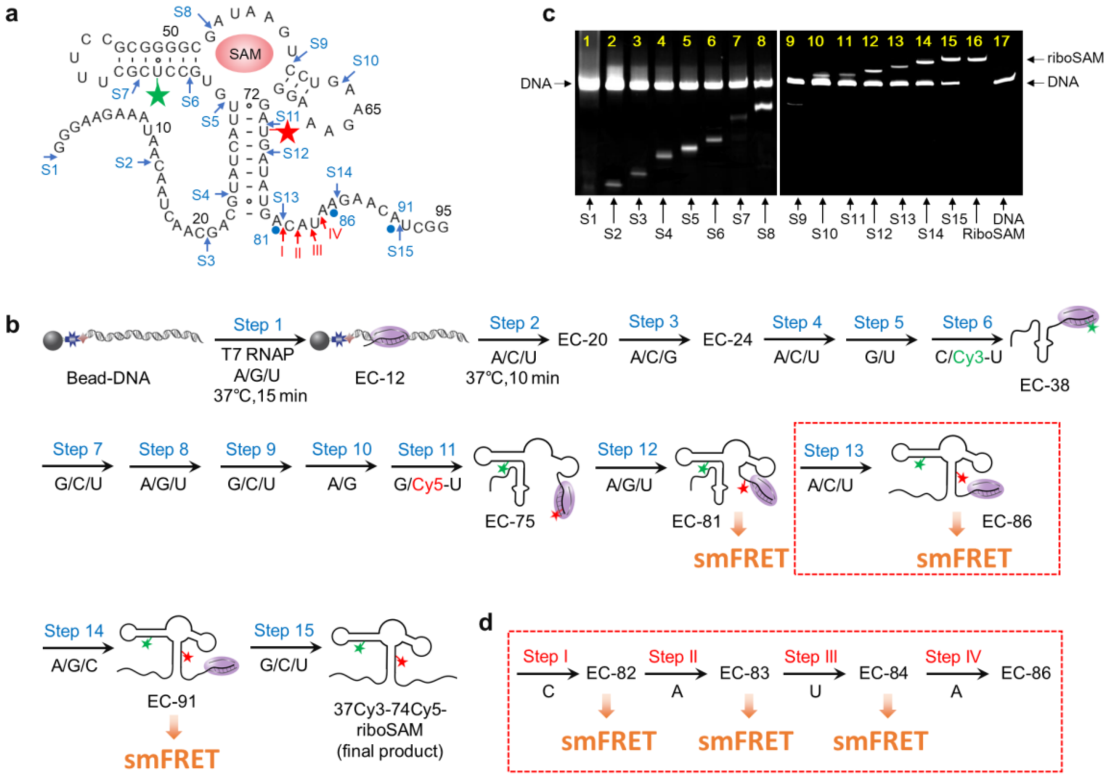

Supplementary Figure 3 | The schematic procedure of preparing ECs for co-transcriptional study of riboSAM. a, The secondary structure of riboSAM, with restart sites at each step marked by blue arrows. The pause sites of steps 12, 13 and 14 are marked by blue dots. The fluorophores, Cy3 (green star) and Cy5 (red star) are labeled at sites 37 and 74, respectively. **b,** 15-step reaction for preparing ECs for co-transcriptional study of riboSAM. The EC-81, EC-86 and EC-91 dissociated from the beads in steps 12, 13, and 14 were measured by smFRET. The reagent usages for the 15-step reaction are listed in Supplementary Table 4. **c,** Gel shift assays of the nascent RNA in the active transcription. The liquid phase removed by SPE at steps 1 to 15 was loaded at Lanes 1 to 15. The standard samples, riboSAM and DNA templates were loaded at Lanes 16 and 17, respectively. The experiment was repeated independently for at least three times. **d,** Elongation from EC-81 to EC-86 was also performed by steps I, II, III and IV.

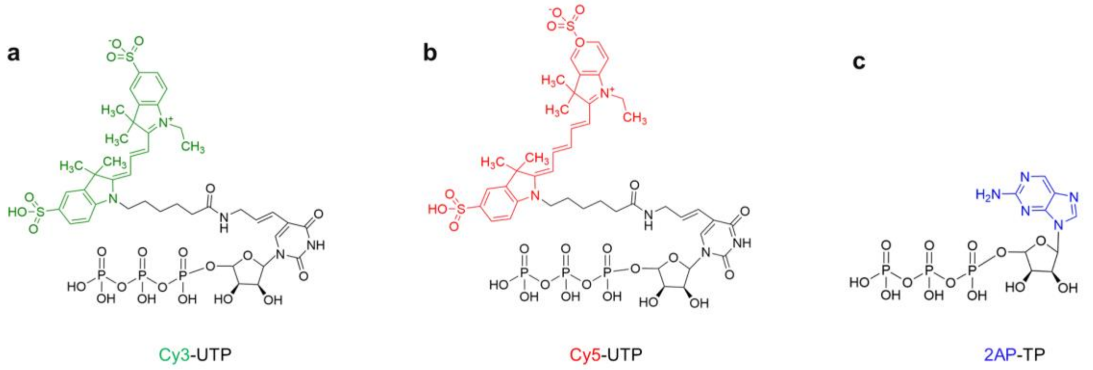

Supplementary Figure 4 │ The chemical structures of Cy3-UTP (a), Cy5-UTP (b) and 2AP triphosphate, 2AP-TP (c).

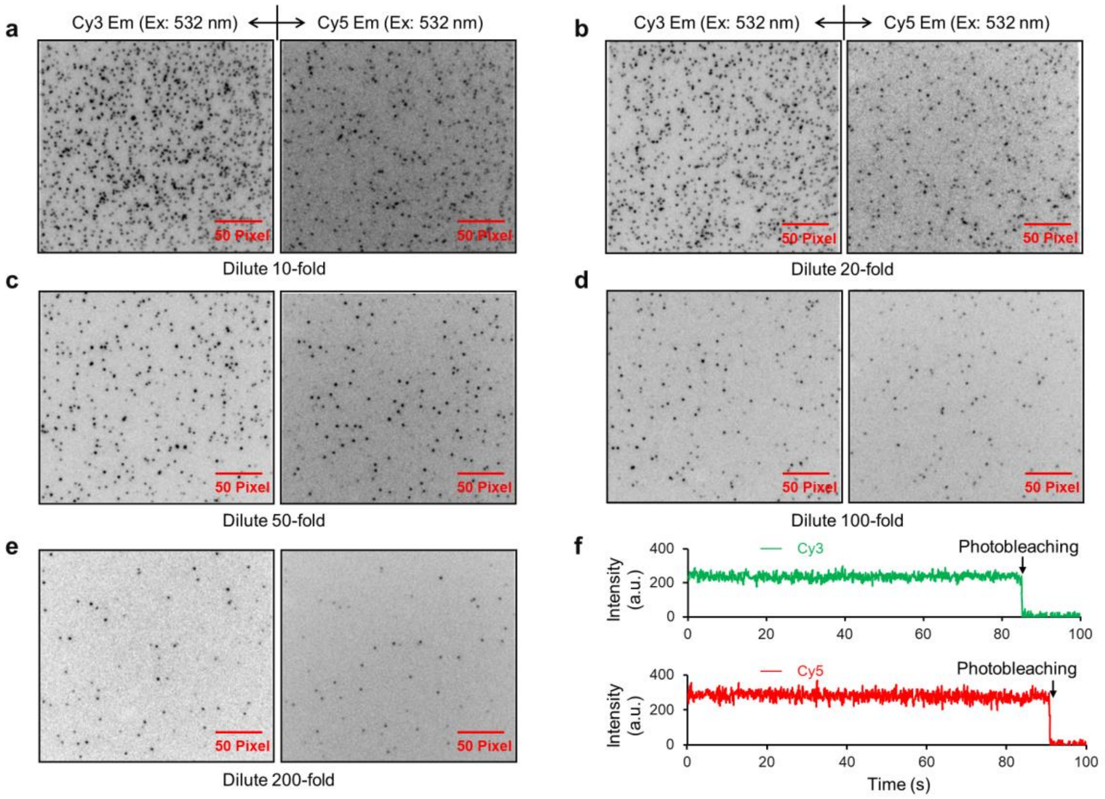

Supplementary Figure 5 │ smFRET measurements of the ECs after dissociation from beads. a-e, The images of surface-immobilized ECs after dilution 10-fold (**a**), 20-fold (**b**), 50-fold (**c**), 100-fold (**d**) and 200-fold (**e**), respectively. The experiment was repeated independently for at least three times. **f,** The photobleaching events of the donor (Cy3) and acceptor (Cy5) in a EC were observed with the excitation wavelength at 532 nm (green curve) and 640 nm (red curve), respectively.

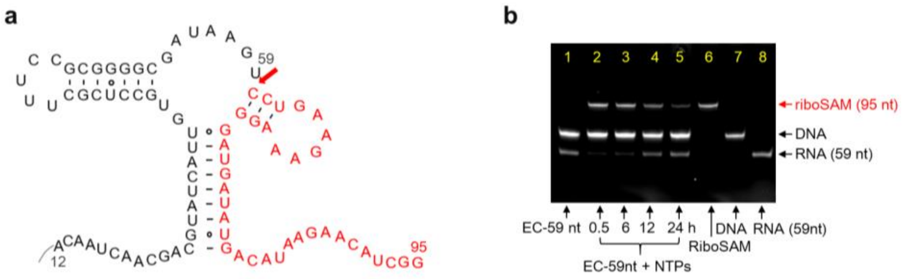

Supplementary Figure 6 │ The processive activity of EC-59 after dissociation from beads. a, The secondary structure of the full-length riboSAM, containing the transcript in EC-59 (in black) and the nucleotides to be transcribed (in red) in the experiments of processive activity. **b,** Processive activity of EC-59 after dissociation from beads for 0.5-24 hours. Full-length riboSAM were generated by adding NTPs to EC-59 after dissociation from beads for 0.5 h (Lane 2), 6 h (Lane 3), 12 h (Lane 4) and 24 h (Lane 5). Lanes 6, 7 and 8 contained full-length riboSAM, DNA template and the 59 nt RNA as control, respectively. The experiment was repeated independently for at least three times. The detailed procedure and reagent usages for testing processive activity of EC-59 are listed in Supplementary Table 5.

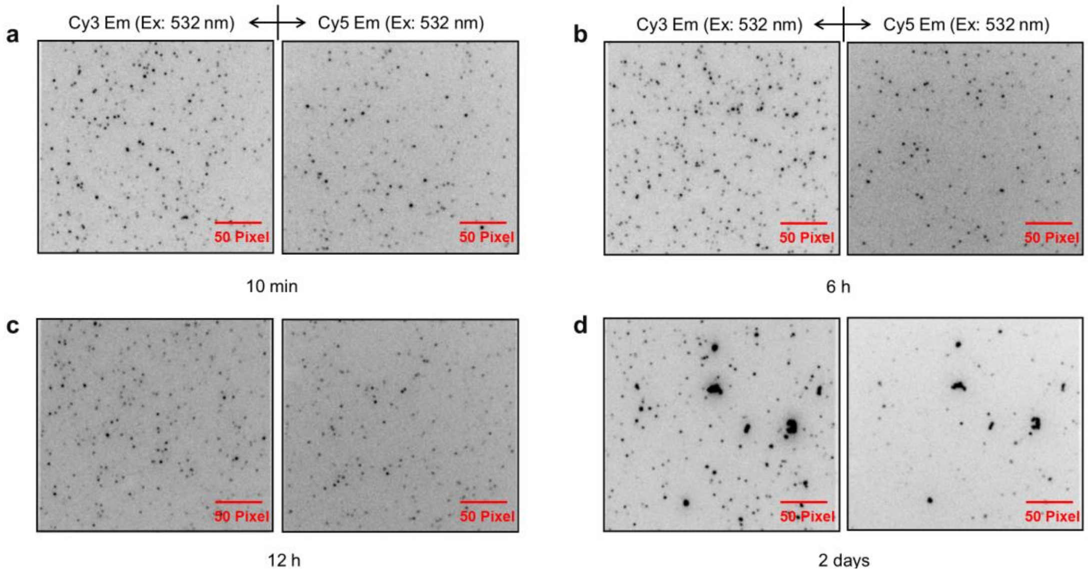

Supplementary Figure 7 │ Images of surface-immobilized ECs after dissociation from the solid-phase beads for 10 min (a), 6 h (b), 12 h (c) and 2 days (d). The experiment was repeated independently for at least three times.

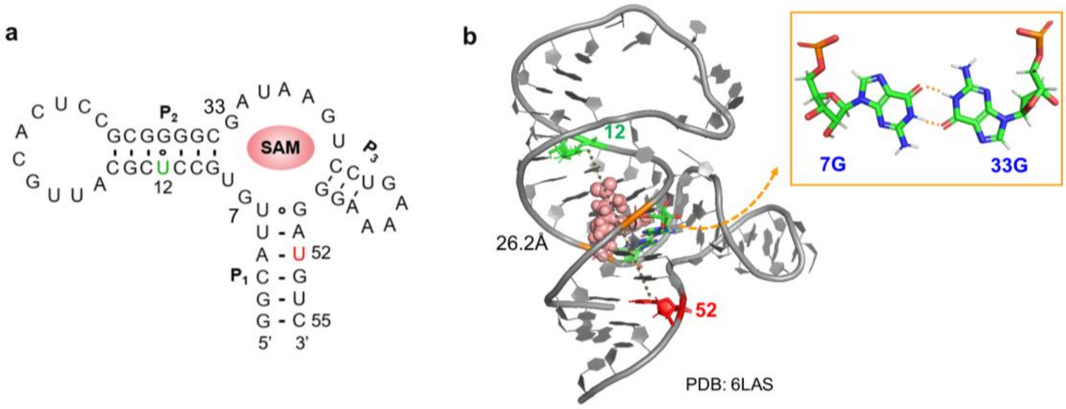

Supplementary Figure 8 │ The crystal structure of the *holo* state of riboSAM. a, The secondary structure of riboSAM used to obtain the crystal structure shown in (**b**). **b,** The crystal structure of riboSAM bound with SAM (PDB ID: 6LAS [https://www.rcsb.org/structure/6LAS]). U12 and U52 are shown in green and red, respectively. The ligand, SAM is shown as pink balls. U12 (corresponds to U37 in our study, in green) is apart from U52 (corresponds to U74 in our study, in red) about 26 Å. A G-G mismatch between 7G and 33G (correspond to 32G and 53G in our study) is zoomed in the orange box.

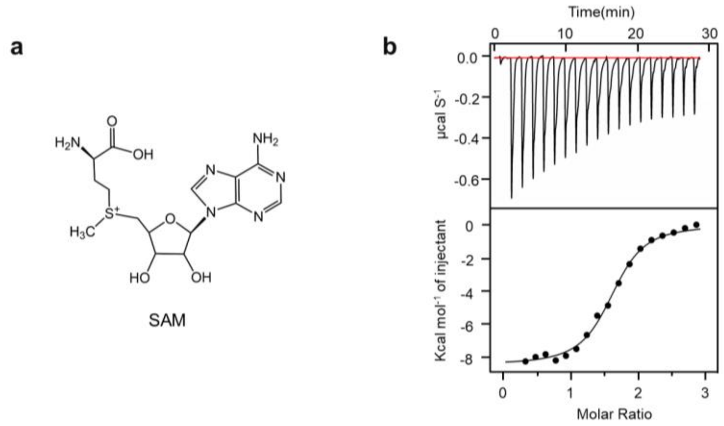

Supplementary Figure 9 │ ITC thermogram of riboSAM titrated against SAM. a, The chemical structure of SAM. **b,** ITC curves of 45 µM riboSAM upon the titration of SAM. SAM bound to riboSAM with K_d of 1.03 ± 0.23 µM.

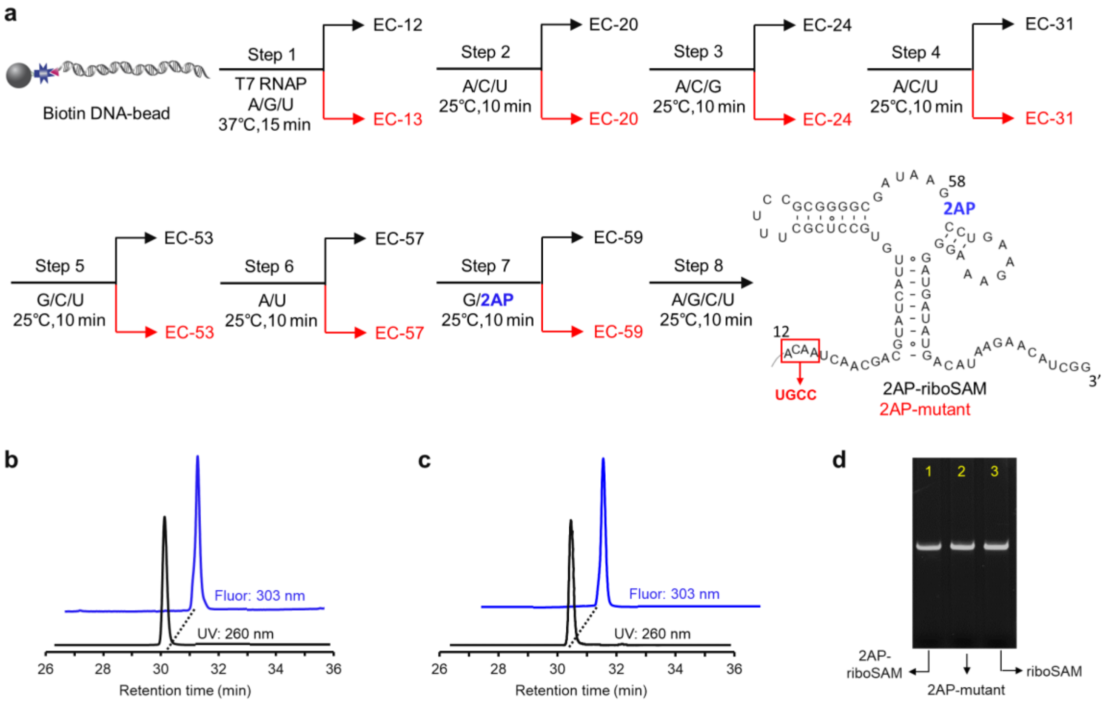

Supplementary Figure 10 | The schematic procedure of producing 2AP-riboSAM and 2AP-mutant. a, The schematic procedure of 8-step reaction for producing 2AP-riboSAM (following the black arrows) and 2AP-mutant (following the red arrows) labeled with 2AP (in blue) at site 59. The mutated nucleotides are shown in red. The detailed procedure and reagent usages for the syntheses are listed in Supplementary Table 6. **b,** The HPLC curves of 2AP-riboSAM with 260 nm UV irradiation (in black) and 303 nm fluorescence excitation (in blue). **c,** The HPLC curves of 2AP-mutant with 260 nm UV irradiation (in black) and 303 nm fluorescence excitation (in blue). **d,** Denaturing PAGE image of the purified 2AP-riboSAM (Lane 1), 2AP-mutant (Lane 2) and the unlabeled riboSAM (Lane 3) irradiated under 260 nm UV. The experiment was repeated independently for at least three times.

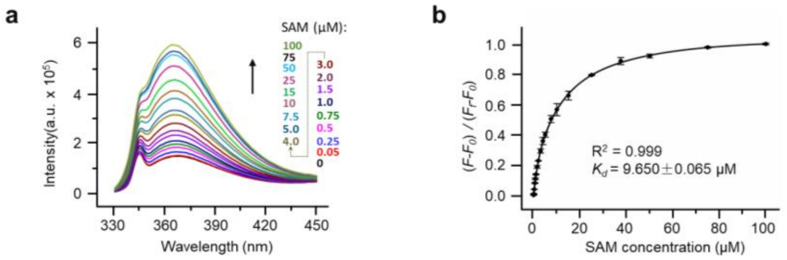

Supplementary Figure 11 │ The steady-state fluorescence spectra of 2AP-riboSAM upon the titration of SAM. a, The fluorescence spectra of 0.5 μM 2AP-riboSAM mixing with 0–200 folds of SAM (SAM concentrations increase from bottom to top). **b,** The normalized fluorescence change of 2AP-riboSAM is plotted as a function of SAM concentration, yielding the dissociation constant K_d. The data are presented as mean values ± s.d. for at least three independent experiments.

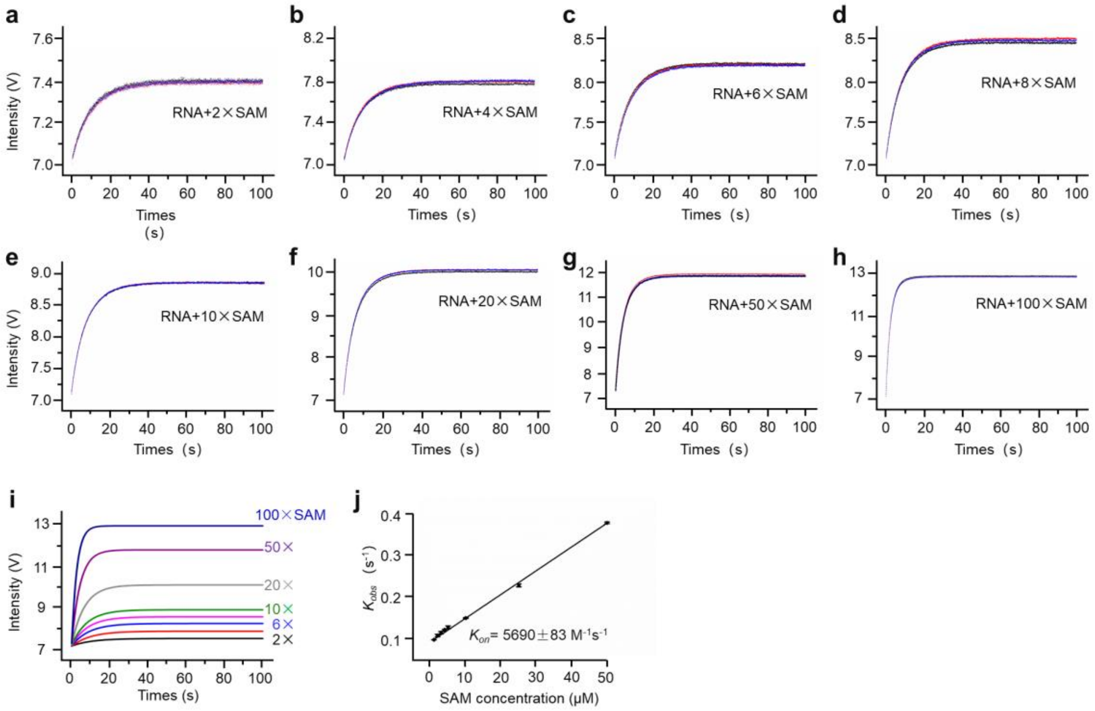

Supplementary Figure 12 │ The stopped-flow trajectories of 0.5 μM 2AP-riboSAM rapidly mixing with 2- (a), 4- (b), 6- (c), 8- (d), 10- (e), 20- (f), 50- (g) and 100-fold SAM (h). i, The superposed stopped-flow curves of 2AP-riboSAM mixing with 2–100 folds of SAM. **j,** The observed rate constants, K_{obs} are plotted as a function of SAM concentration, with the slope yielding the association rate constant K_{on} of 5690 ± 83 $M^{-1}s^{-1}$. The data are presented as mean values ± s.d. for three independent experiments. K_{obs} of 2AP-riboSAM at different concentrations of SAM are listed in Supplementary Table 16.

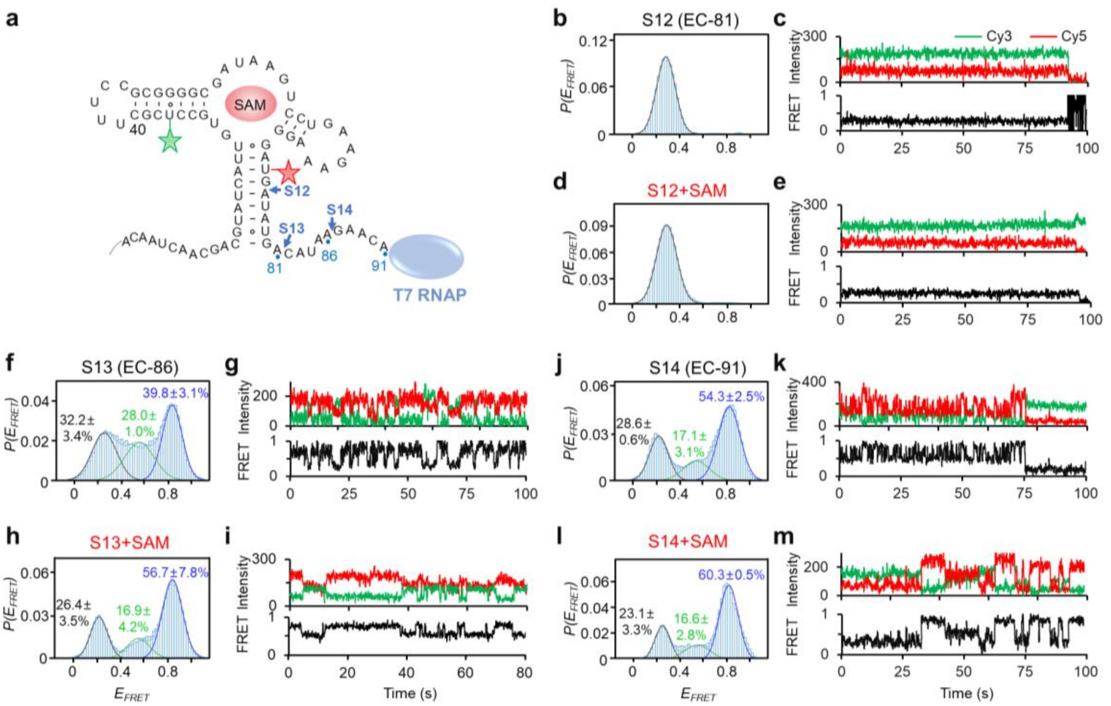

Supplementary Figure 13 │ smFRET results of EC-81, EC-86 and EC-91 of 37Cy3-74Cy5-riboSAM. a, The secondary structure of riboSAM labeled with Cy3 (green star) and Cy5 (red star) at sites 37 and 74, respectively. The T7 RNAP is shown as blue sphere. The transcriptional restart sites in steps 12, 13 and 14 are marked by blue arrows. The reagent usages are listed in Supplementary Table 4. **b-m,** smFRET histograms and trajectories of EC-81, EC-86 and EC-91 in the absence and presence of SAM. Mean values ±s.d. of triplicate experiments are shown for peak percentages.

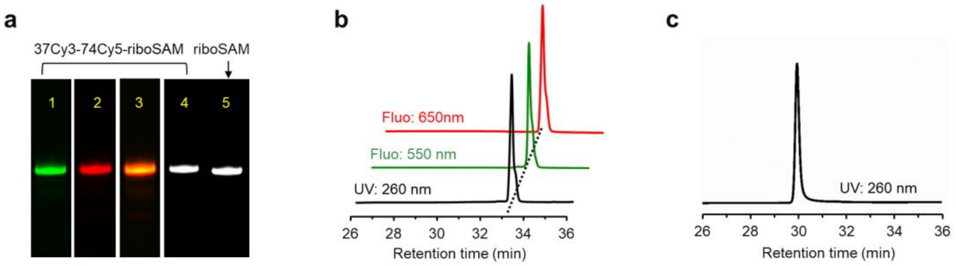

Supplementary Figure 14 │ PAGE images and HPLC curves of the purified 37Cy3-74Cy5-riboSAM. a, Denaturing PAGE images of 37Cy3-74Cy5-riboSAM irradiated under 530 nm fluorescence (Lane 1), 620 nm fluorescence (Lane 2) and 260 nm UV (Lane 4). Lane 3 is the merged image of Lanes 1 and 2. The unlabeled riboSAM was loaded at Lane 5 as control. The experiments were repeated independently for at least three times. **b,** The HPLC curves of 37Cy3-74Cy5-riboSAM with 260 nm UV irradiation (in black), 550 nm fluorescence excitation (in green) and 650 nm fluorescence excitation (in red). **c,** The HPLC curve of unlabeled riboSAM with 260 nm UV irradiation.

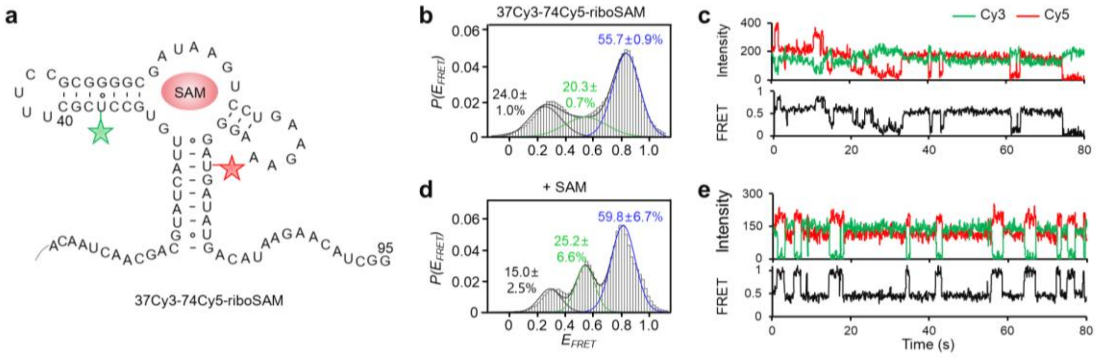

Supplementary Figure 15 │ The smFRET results of the full-length 37Cy3-74Cy5-riboSAM. a, The secondary structure of 37Cy3-74Cy5-riboSAM labeled with Cy3 (green star) and Cy5 (red star) at sites 37 and 74, respectively. The detailed reagent usages are listed in Supplementary Table 7. smFRET histogram and trajectory of 37Cy3-74Cy5-riboSAM in the absence of SAM (**b** and **c**) and presence of SAM (**d** and **e**). Mean values ± s.d. of triplicate experiments are shown for peak percentages.

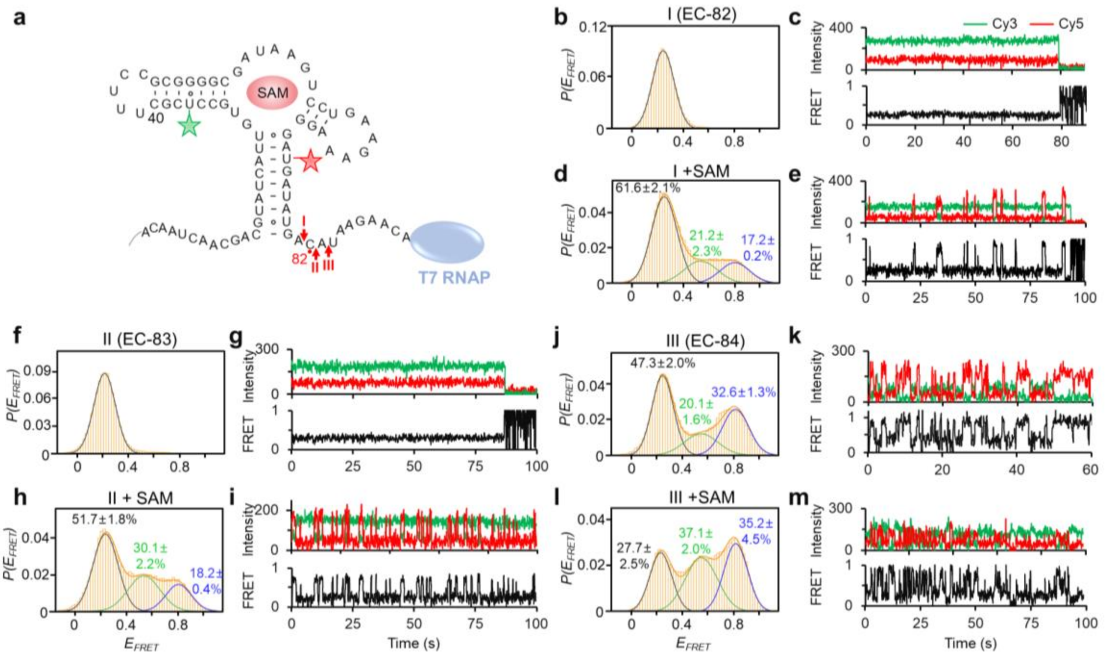

Supplementary Figure 16 │ smFRET results of EC-82, EC-83 and EC-84 of riboSAM. a, The secondary structure of 37Cy3-74Cy5-riboSAM labeled with Cy3 (green star) and Cy5 (red star) at sites 37 and 74, respectively. The T7 RNAP is shown as a blue sphere. The transcriptional restart sites in steps I, II and III are marked by red arrows. The reagent usages are listed in Supplementary Table 4. **b-m,** smFRET histograms and trajectories of EC-82, EC-83 and EC-84 in the absence and presence of SAM. Mean values ± s.d. of triplicate experiments are shown for peak percentages.

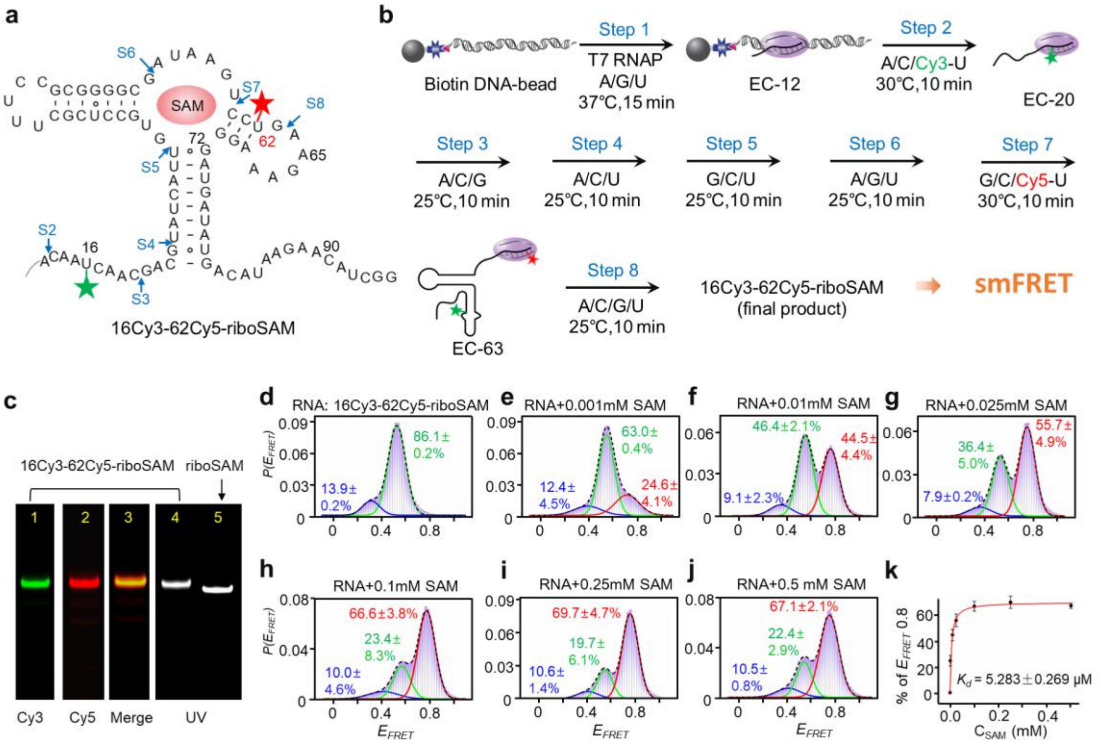

Supplementary Figure 17 │ smFRET results for the full-length 16Cy3-62Cy5-riboSAM. a, The secondary structure of 16Cy3-62Cy5-riboSAM labeled with Cy3 (green star) and Cy5 (red star) at sites 16 and 62, respectively. 16Cy3-62Cy5-riboSAM was synthesized by a 8-step reaction, with the transcriptional restart sites marked by blue arrows. **b,** The schematic procedure of the 8-step reaction for producing 16Cy3-62Cy5-riboSAM. The detailed reagent usages are listed in Supplementary Table 8. **c,** Denaturing PAGE images of the purified 16Cy3-62Cy5-riboSAM irradiated under 530 nm fluorescence (Lane 1), 620 nm fluorescence (Lane 2) and 260 nm UV (Lane 4). Lane 3 is the merged image of Lanes 1 and 2. The unlabeled riboSAM was loaded at Lane 5 as control. The experiments were repeated independently for at least three times. **d-j,** smFRET histograms of 16Cy3-62Cy5-riboSAM at 2mM Mg^{2+} and 0–0.5 mM SAM. **k**, The proportions of the high-FRET peak (E_{FRET} ~0.8) are plotted as a function of SAM concentration, yielding the dissociation constant K_d. Mean values ± s.d. of triplicate experiments are shown for peak percentages.

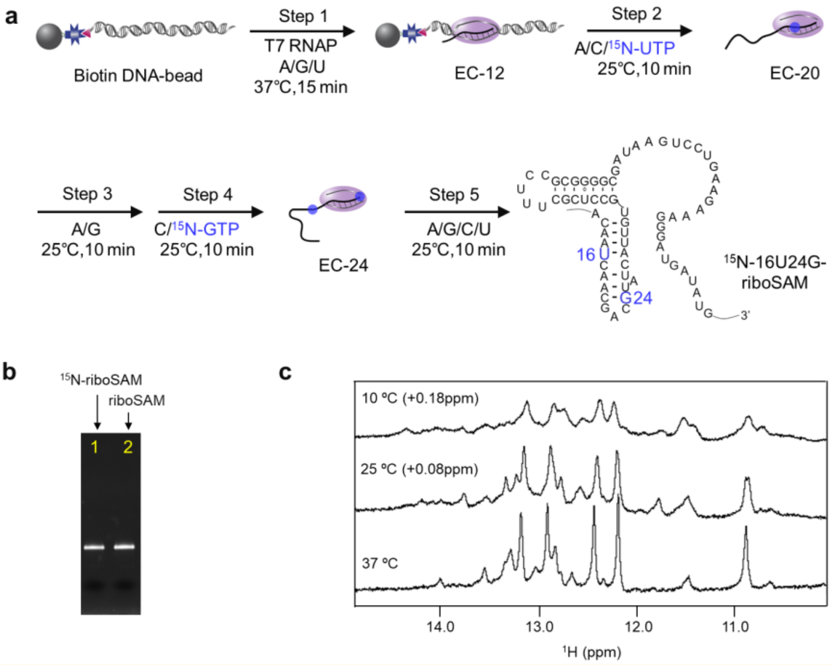

Supplementary Figure 18 | NMR measurements for the position-specific isotopically labeled ^{15}N-16U24G-riboSAM. a, The schematic procedure of 5-step reaction for producing ^{15}N-16U24G-riboSAM labeled with ^{15}N at sites 16 and 24 (in blue). The detailed reagent usages are listed in Supplementary Table 9. **b,** Denaturing PAGE image of the purified ^{15}N-16U24G-riboSAM (Lane 1) and the unlabeled riboSAM (Lane 2) irradiated under 260 nm UV. The experiment was repeated independently for at least three times. **c,** The ^{1}H-1D NMR spectra of ^{15}N-16U24G-riboSAM at 2mM Mg^{2+} at 10 ℃, 25 ℃ and 37 ℃, respectively.

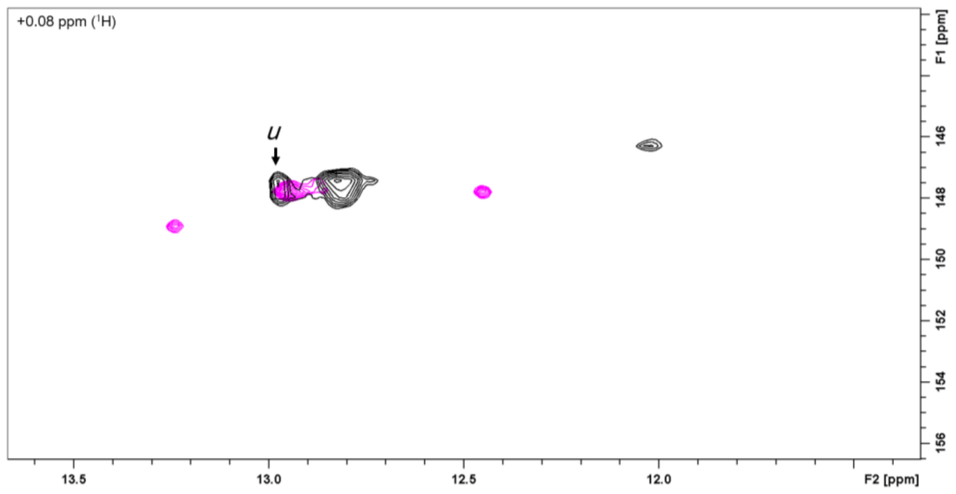

Supplementary Figure 19 │ The HSQC spectra of 0.5 mM ^{15}N-16U24G-riboSAM in the absence (magenta) and presence of 2 mM Mg^{2+} (black). The superposed peak *u* (marked by a arrow) is speculated to be from a unfolded structure.

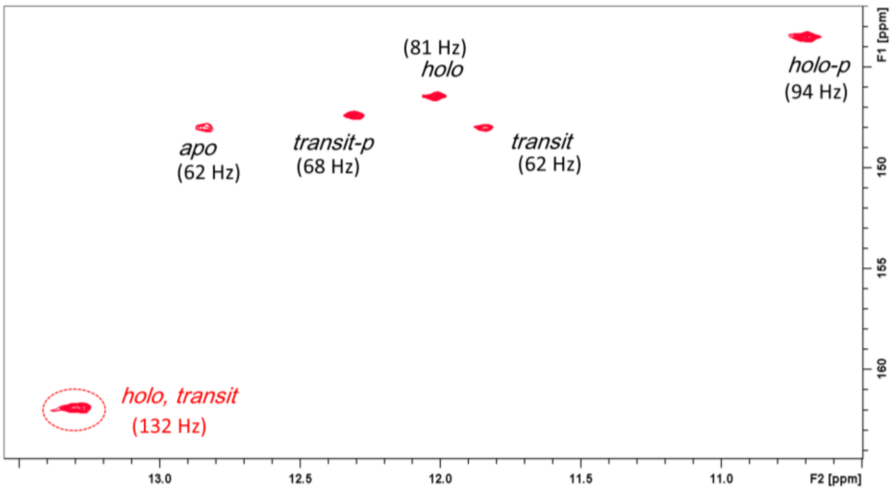

Supplementary Figure 20 | The HSQC spectrum of 0.15 mM ^{15}N-25U32G-riboSAM in the presence of 2 mM Mg^{2+} and 0.15 mM SAM. The peak width of the peaks in red circle is ~132 Hz, much wider than other peaks (~62-94 Hz), indicating the overlap of two peaks with very close chemical shifts.

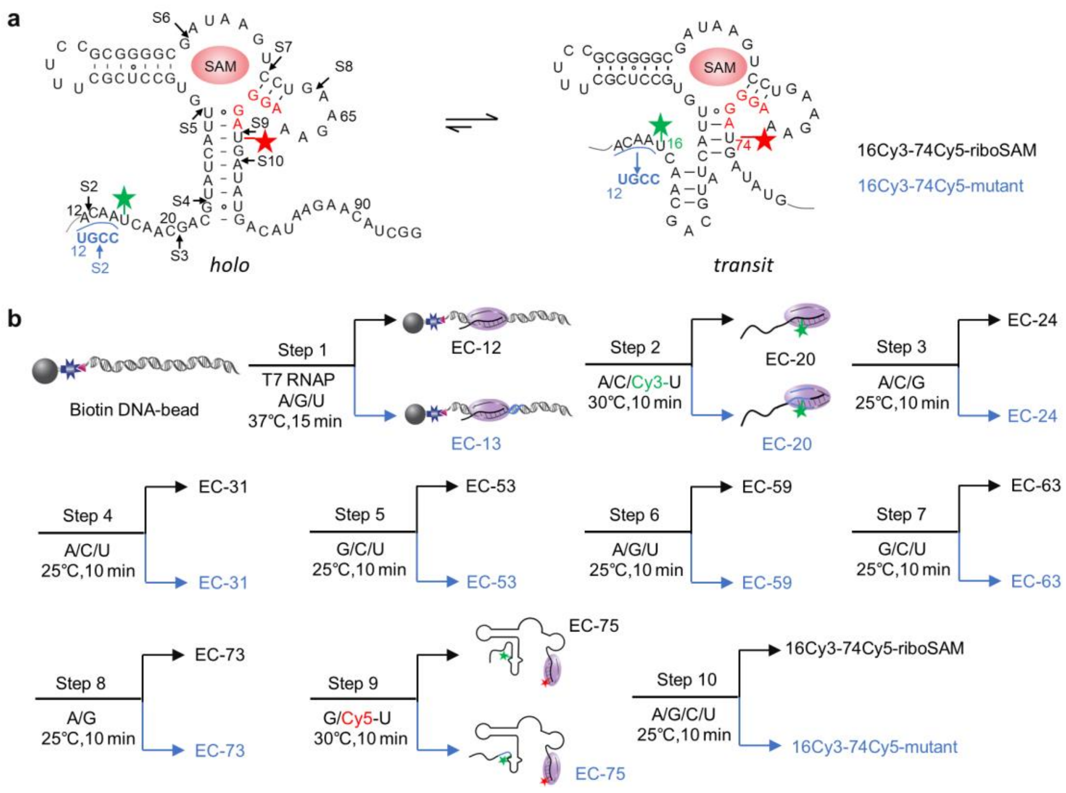

Supplementary Figure 21 │ The schematic procedure of producing 16Cy3-74Cy5-riboSAM and 16Cy3-74Cy5-mutant. a, The secondary structures of 16Cy3-74Cy5-riboSAM and 16Cy3-74Cy5-mutant labeled with Cy3 (green star) and Cy5 (red star) at sites 16 and 74, respectively. The transcriptional restart sites are marked by black arrows, except step 2 in the mutant is marked by blue arrow. The mutated nucleotides are shown in blue. **b,** The schematic procedure of 10-step reaction for producing 16Cy3-74Cy5-riboSAM (following the black arrows) and 16Cy3-74Cy5-mutant (following the blue arrows). The detailed procedure and reagent usages for the syntheses are listed in Supplementary Table 11.

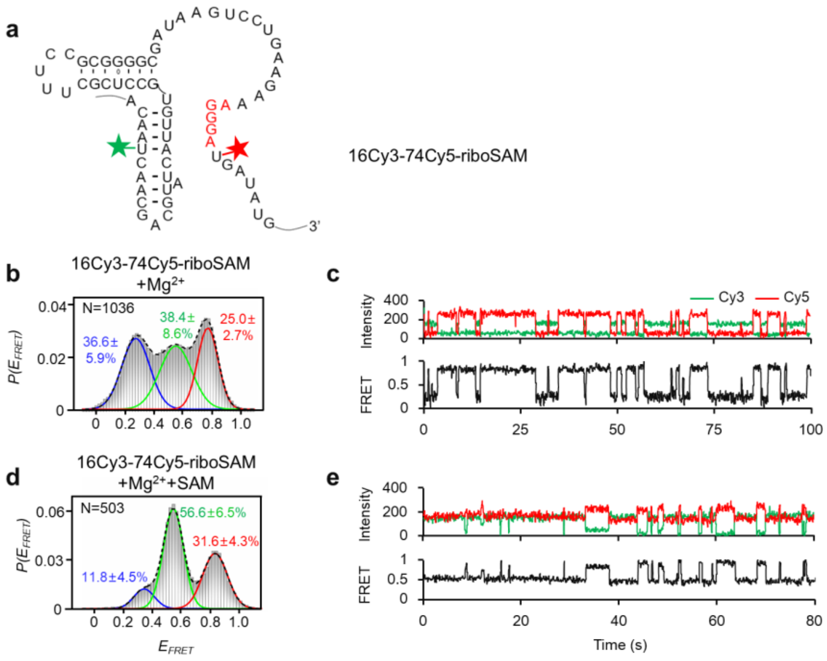

Supplementary Figure 22 │ smFRET results for 16Cy3-74Cy5-riboSAM in the absence and presence of SAM. a, The secondary structure of 16Cy3-74Cy5-riboSAM labeled with Cy3 (green star) and Cy5 (red star) at sites 16 and 74, respectively. **b-e,** smFRET histograms and trajectories of 16Cy3-74Cy5-riboSAM in the absence (**b** and **c**) and presence of SAM (**d** and **e**). Mean values ± s.d. of triplicate experiments are shown for peak percentages.

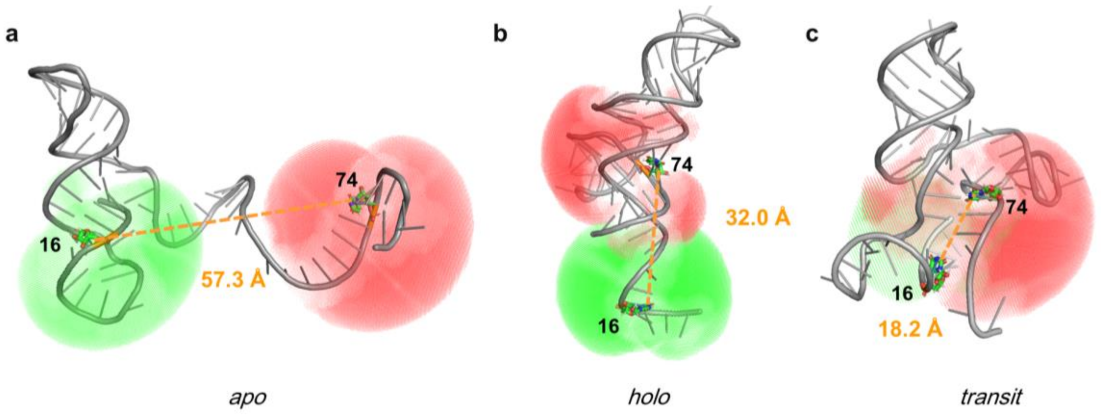

Supplementary Figure 23 │ The simulated *apo* (a), *holo* (b) and *transit* (c) structures of riboSAM with Cy3-Cy5 labels at sites 16 and 74. The distances between 16 and 74 (shown in sticks) in *apo*, *holo* and *transit* are 57.3, 32.0 and 18.2 Å, matching the experimental and FPS-calculated E_{FRET}. Cy3 and Cy5 accessible volume (AV) clouds are shown in green and red spheres, respectively. The parameters and results of FPS software are listed in Supplementary Table 13.

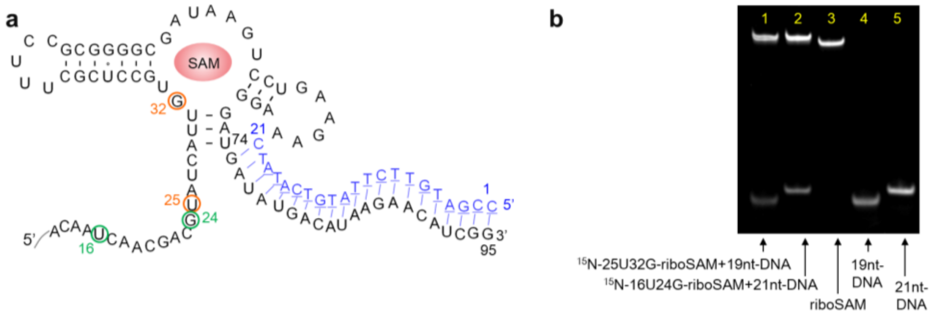

Supplementary Figure 24 │ The hybridization of ^{15}N-labeled riboSAM and short DNAs for NMR study. a, The schematic diagram of hybridization between riboSAM (black) and 19nt-DNA (blue, underlined) or 21nt-DNA (blue). **b,** 15% native PAGE image of the riboSAM-DNA hybrids. The hybrid of ^{15}N-25U32G-riboSAM and 19nt-DNA was loaded at Lane 1, the hybrid of ^{15}N-16U24G-riboSAM and 21nt-DNA was loaded at Lane 2. Lanes 3, 4 and 5 contained riboSAM, 19nt-DNA and 21nt-DNA as control, respectively. The experiment was repeated independently for at least three times.

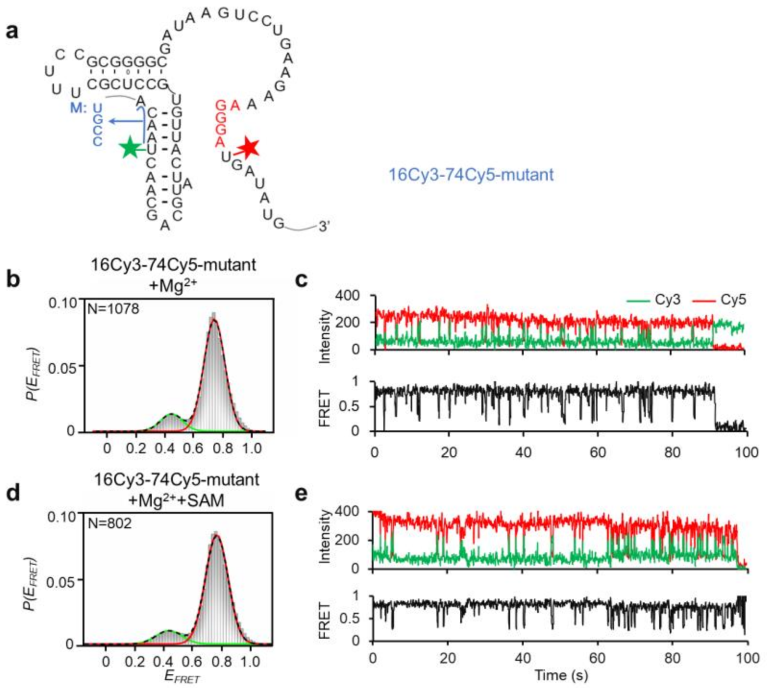

Supplementary Figure 25 │ smFRET results of 16Cy3-74Cy5-mutant in the absence and presence of SAM. a, The secondary structure of 16Cy3-74Cy5-mutant labeled with Cy3 (green star) and Cy5 (red star) at sites 16 and 74, respectively. The mutated nucleotides are in blue. **b-e,** smFRET histograms and trajectories of 16Cy3-74Cy5-mutant in the absence (**b** and **c**) and presence of SAM (**d** and **e**).

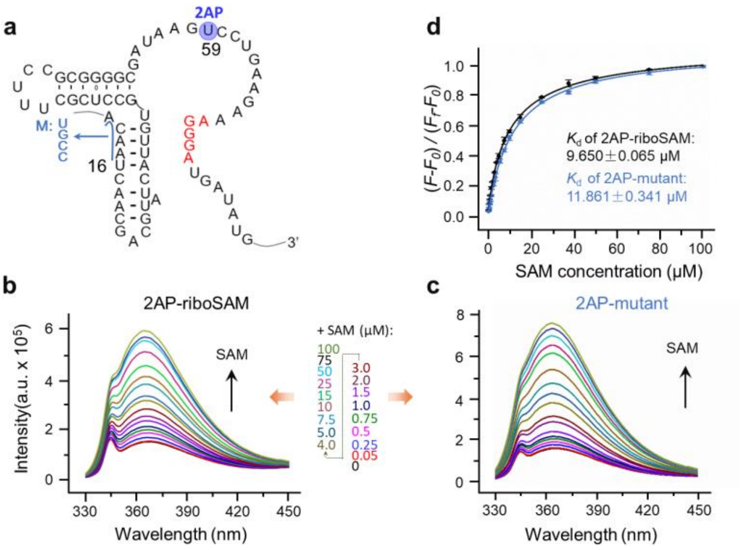

Supplementary Figure 26 │ The steady-state fluorescence spectra of 2AP-riboSAM and 2AP-mutant upon the titration of SAM. a, The secondary structure of 2AP-riboSAM and 2AP-mutant, labeled with 2AP (in blue) at site 59. The mutated nucleotides are shown in blue. The RBS is shown in red. **b,** The fluorescence spectra of 0.5 μM 2AP-riboSAM mixing with 0–200 folds of SAM (SAM concentrations increase from bottom to top). **c,** The fluorescence spectra of 0.5 μM 2AP-mutant mixing with 0–200 folds of SAM. **d,** The normalized fluorescence change of 2AP-riboSAM and 2AP-mutant are plotted as a function of SAM concentration, yielding the dissociation constant K_d of 9.650 ± 0.065 and 11.861 ± 0.341 μM, respectively. The data are presented as mean values ± s.d. for at least three independent experiments.

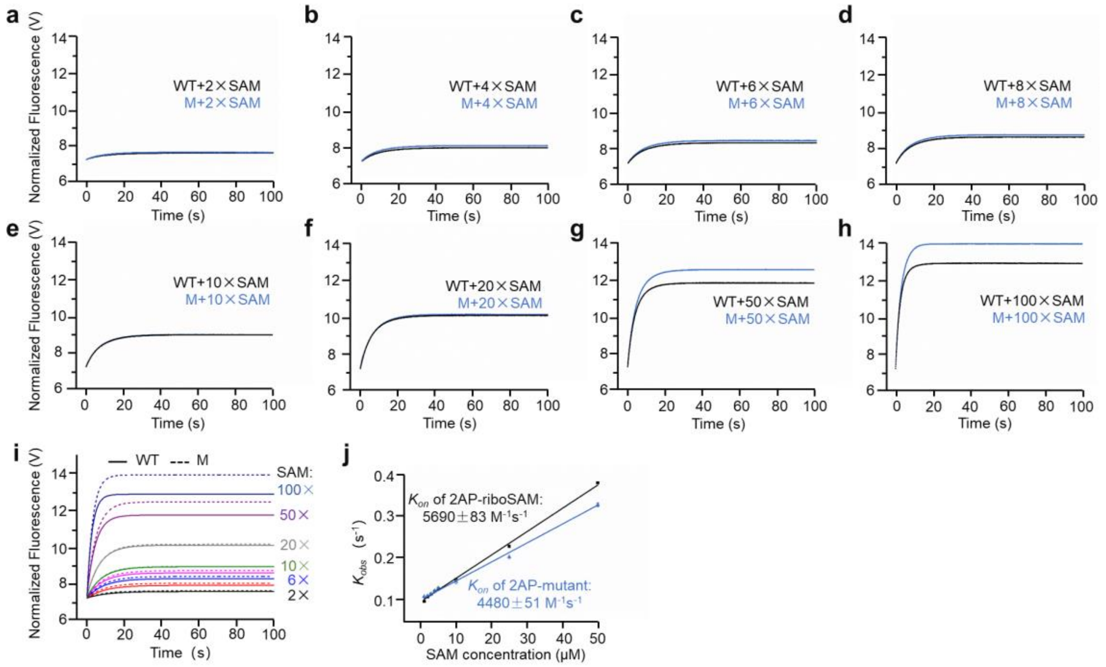

Supplementary Figure 27 │ The stopped-flow trajectories of 0.5 μM 2AP-riboSAM (in black) and 2AP-mutant (in blue) rapidly mixing with 2- (a), 4- (b), 6- (c), 8- (d), 10- (e), 20- (f), 50- (g) and 100-fold (h) of SAM. i, The superposed stopped-flow curves of 2AP-riboSAM (solid lines) and 2AP-mutant (dotted lines) mixing with 2–100 folds of SAM. **j,** K_{obs} of 2AP-riboSAM and 2AP-mutant are plotted as a function of SAM concentration, with the slope yielding the association rate constant K_{on} of 5690 ± 83 and 4480 ± 51 $M^{-1}s^{-1}$, respectively. The data are presented as mean values ± s.d. for three independent experiments.

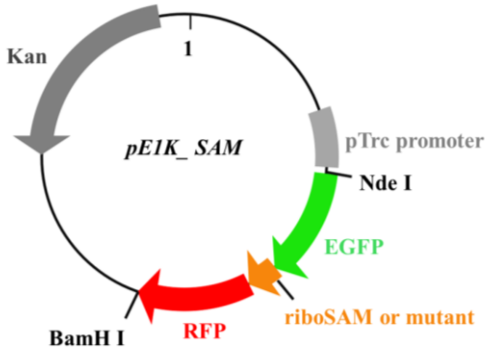

Supplementary Figure 28 │ The plasmid construct for cellular translation assays of riboSAM. The sequences of riboSAM or its mutant (in orange) are located downstream of the reporter EGFP (in green) and upstream of the reporter RPF (red) in *pE1K_SAM* plasmid.

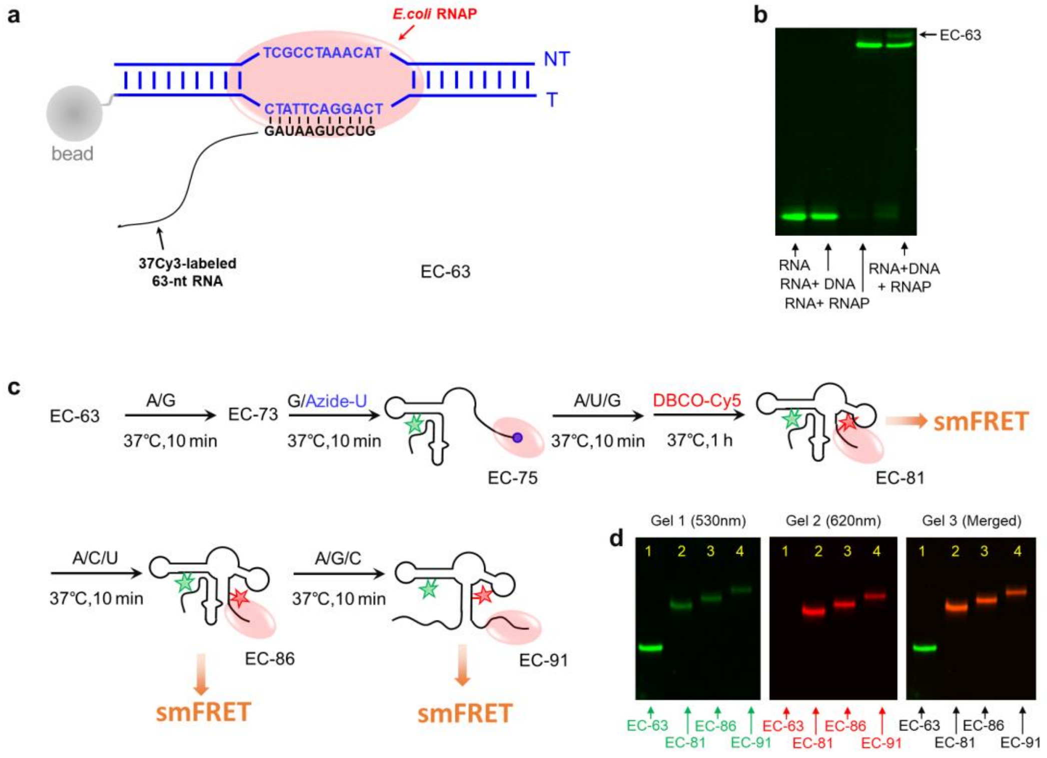

Supplementary Figure 29 │ The schematic procedure of preparing ECs containing a dual-labeled RNA and *E. coli* RNAP. a, The schematic diagram of assembling ECs containing a 63-nt RNA labeled with Cy3 at site 37, DNA template (blue lines) and *E. coli* RNAP (red sphere). **b,** 6% native PAGE image of 63-nt RNA (Lane 1), mixture of RNA and DNA template (Lane 2), mixture of RNA and *E. coli* RNAP (Lane 3), mixture of RNA, DNA and *E. coli* RNAP (Lane 4) irradiated under 530 nm fluorescence. **c,** The schematic procedure of producing EC-81, EC-86 and EC-91 with *E. coli* RNAP for smFRET study. The Cy3 and Cy5 labels are shown as green and red stars, respectively. The detailed reagent usages are listed in Supplementary Tables 14 and 15. **d,** Gel shift assays of the nascent RNA in ECs with *E. coli* RNAP irradiated under 530 nm fluorescence (Left gel) and 620 nm fluorescence (middle gel). The right gel is the merged image of the left and middle gels. The experiment was repeated independently for at least three times.

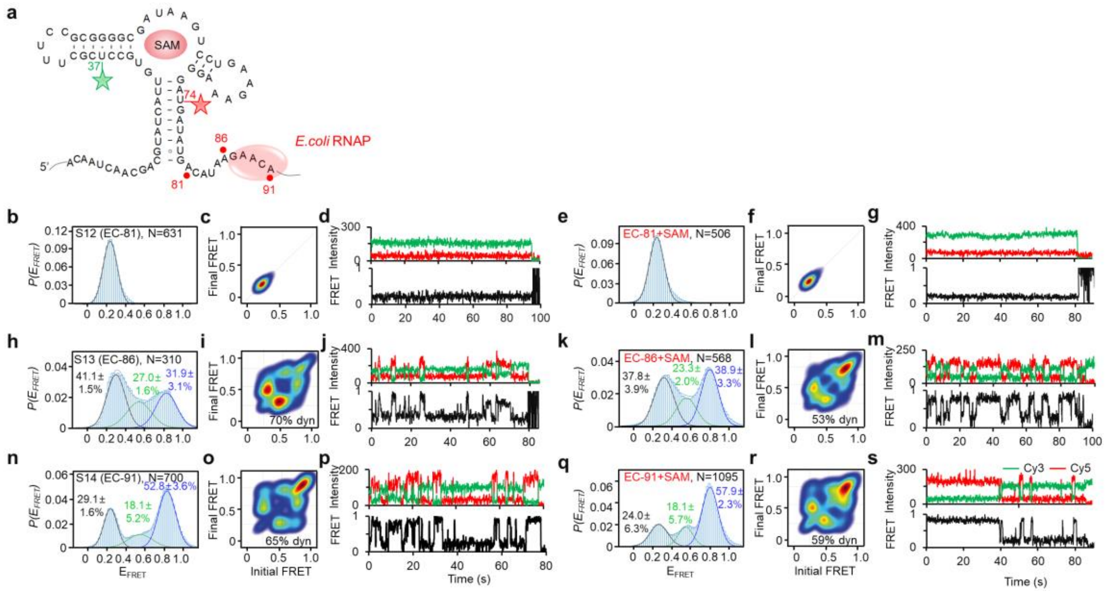

Supplementary Figure 30 │ smFRET results of EC-81, EC-86 and EC-91 with *E. coli* RNAP. a, The secondary structure of riboSAM labeled with Cy3 (green star) and Cy5 (red star) at sites 37 and 74, respectively. The *E. coli* RNAP is shown as red sphere. The transcriptional pause sites in EC-81, EC-86 and EC-91 are marked by red dots. **b-s**, smFRET histograms, transition density plots and trajectories of EC-81, EC-86 and EC-91 in the absence and presence of SAM.

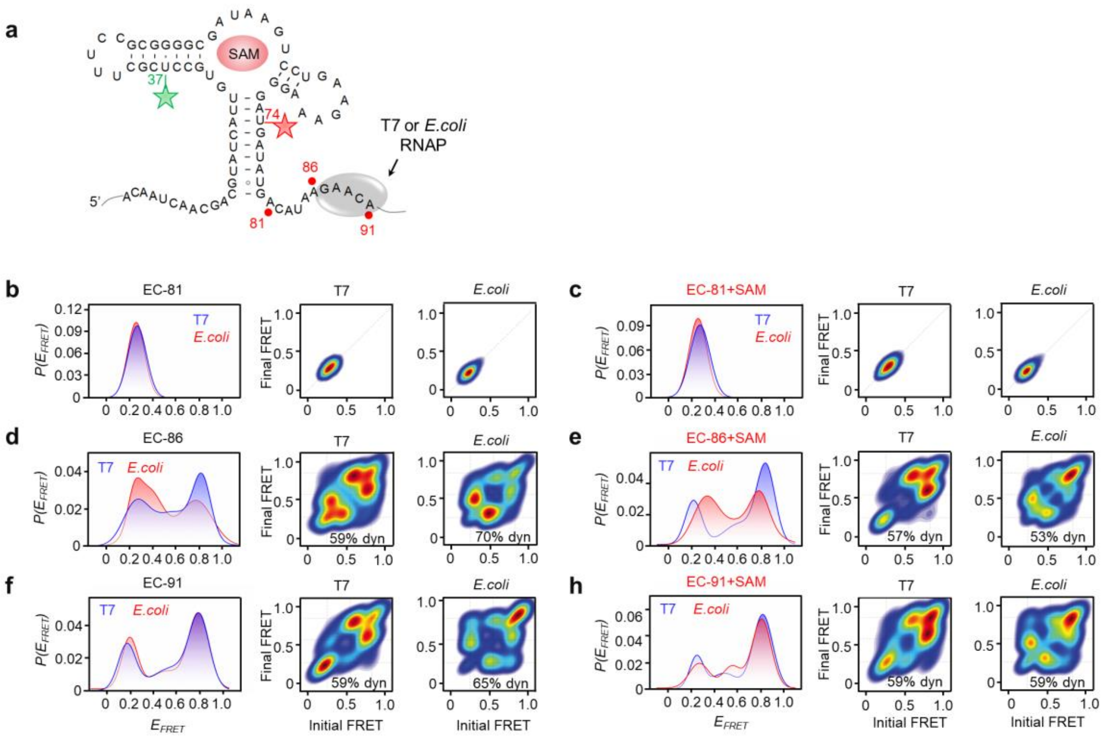

Supplementary Figure 31 │ Comparison of smFRET results for EC-81, EC-86 and EC-91 containing T7 vs. *E. coli* RNAP. a, The secondary structure of riboSAM labeled with Cy3 (green star) and Cy5 (red star) at sites 37 and 74. T7 or *E. coli* RNAP is shown as grey sphere. The transcriptional pause sites in EC-81, EC-86 and EC-91 are marked by red dots. **b-h,** The superposition of smFRET histograms of EC-81, EC-86 and EC-91 with T7 (blue) or *E. coli* RNAP (red), and transition density plots in the absence and presence of SAM.

Supplementary Table 1 │ The DNA/RNA sequences used for producing ECs using T7 RNAP for riboSAM structural study.

DNA/RNA	Sequence
riboSAM	5′-GGGAAGAAAUAACAAUCAACGACGUAUCAUUGUGCCUCGCUUUCCGCGGGGCGAUAAGUCCUGAAGAAAGGGAUGAUAUGACAUAAGAACAUCGG
DNA template for riboSAM	5′-mCmCGATGTTCTTATGTCATATCATCCCTTTCTTCAGGACTTATCGCCCCGCGGAAAGCGAGGCACAATGATACGTCGTTGATTGTTATTTCTTCCCTATAGTGAGTCGTATTA*TGGACTAGCTGAATCAGA*
DNA non-template for riboSAM	5′-Desthiobiotin-*TCTGATTCAGCTAGTCCA*TAATACGACTCACTATAGGGAAGAAATAACAATCAACGACGTATCATTGTGCCTCGCTTTCCGCGGGGCGATAAGTCCTGAAGAAAGGGATGATATGACATAAGAACATCGG

The T7 promoter sequences in DNAs are underlined, and the linker sequences (italic) are inserted in DNA to alleviate potential steric hindrance between beads and T7 RNAP during transcription. mC represents 2′-O-methyl-dC.

Supplementary Table 2 | The DNA/RNA sequences used for structural study of isolated riboSAM.

DNA/RNA	Sequence
riboSAM	5′-GGGAAGAAAUAACAAUCAACGACGUAUCAUUGUGCCUCGCUUUCCGCGGGGCGAUAAGUCCUGAAGAAAGGGAUGAUAUGACA**UAAGAACAUCGG**
DNA template for riboSAM	5′-mCmCGATGTTCTTATGTCATATCATCCCTTTCTTCAGGACTTATCGCCCCGCGGAAAGCGAGGCACAATGATACGTCGTTGATTGTTATTTCTTCCCTATAGTGAGTCGTATTA*TGGACTAGCTGAATCAGA*
DNA non-template for riboSAM	5′-Biotin-*TCTGATTCAGCTAGTCCA*TAATACGACTCACTATAGGGAAGAAATAACAATCAACGACGTATCATTGTGCCTCGCTTTCCGCGGGGCGATAAGTCCTGAAGAAAGGGATGATATGACATAAGAACATCGG
12-nt DNA for hybridization with riboSAM or mutant in smFRET	5′-Biotin-CCGATGTTCTTA
21-nt DNA for hybridization with ^{15}N-16U24G-riboSAM in NMR	5′-CCGATGTTCTTATGTCATATC
19-nt DNA for hybridization with ^{15}N-25U32G-riboSAM in NMR	5′-CCGATGTTCTTATGTCATA
riboSAM mutant	5′-GGGAAGAAAUAUGCCUCAACGACGUAUCAUUGUGCCUCGCUUUCCGCGGGGCGAUAAGUCCUGAAGAAAGGGAUGAUAUGACA**UAAGAACAUCGG**
DNA template for riboSAM mutant	5′-mCmCGATGTTCTTATGTCATATCATCCCTTTCTTCAGGACTTATCGCCCCGCGGAAAGCGAGGCACAATGATACGTCGTTGAGGCATATTTCTTCCCTATAGTGAGTCGTATTA*TGGACTAGCTGAATCAGA*
DNA non-template for riboSAM mutant	5’-Biotin-*TCTGATTCAGCTAGTCCA*TAATACGACTCACTATAGGGAAGAAATATGCCTCAACGACGTATCATTGTGCCTCGCTTTCCGCGGGGCGATAAGTCCTGAAGAAAGGGATGATATGACATAAGAACATCGG

The T7 promoter sequences in DNAs are underlined, and the linker sequences (italic) are inserted in DNA to alleviate potential steric hindrance between beads and T7 RNAP during transcription. mC represents 2′-O-methyl-dC. The mutated nucleotides are in blue. The bold sequences in riboSAM or its mutant are hybridized with 5’-biotinylated DNA for immobilization in smFRET.

Supplementary Table 3 | The DNA/RNA sequences used for producing 2AP-riboSAM and 2AP-mutant.

DNA/RNA	Sequence
2AP-riboSAM	5′-GGGAAGAAAUAACAAUCAACGACGUAUCAUUGUGC CUCGCUUUCCGCGGGGCGAUAAG**2AP**CCUGAAGAAAG GGAUGAUAUGACAUAAGAACAUCGG
DNA template for 2AP-riboSAM	5′-mCmCGATGTTCTTATGTCATATCATCCCTTTCTTCAG GTCTTATCGCCCCGCGGAAAGCGAGGCACAATGATAC GTCGTTGATTGTTATTTCTTCCCTATAGTGAGTCGTATT A*TGGACTAGCTGAATCAGA*
DNA non-template for 2AP-riboSAM	5′-Biotin-*TCTGATTCAGCTAGTCCA*TAATACGACTCACTAT AGGGAAGAAATAACAATCAACGACGTATCATTGTGCC TCGCTTTCCGCGGGGCGATAAGACCTGAAGAAAGGGA TGATATGACATAAGAACATCGG
2AP-mutant	5′-GGGAAGAAAUAUGCCUCAACGACGUAUCAUUGUGC CUCGCUUUCCGCGGGGCGAUAAG**2AP**CCUGAAGAAAG GGAUGAUAUGACAUAAGAACAUCGG
DNA template for 2AP-mutant	5′-mCmCGATGTTCTTATGTCATATCATCCCTTTCTTCAG GTCTTATCGCCCCGCGGAAAGCGAGGCACAATGATAC GTCGTTGAGGCATATTTCTTCCCTATAGTGAGTCGTATT A*TGGACTAGCTGAATCAGA*
DNA non-template for 2AP-mutant	5’-Biotin-*TCTGATTCAGCTAGTCCA*TAATACGACTCACTAT AGGGAAGAAATATGCCTCAACGACGTATCATTGTGCCT CGCTTTCCGCGGGGCGATAAGACCTGAAGAAAGGGAT GATATGACATAAGAACATCGG

The T7 promoter sequences in DNAs are underlined, and the linker sequences (italic) are inserted in DNAs to alleviate potential steric hindrance between beads and T7 RNAP during transcription. mC represents 2′-O-methyl-dC. The mutated nucleotides are in blue. 2AP is in bold blue.

Supplementary Table 4 | Reagent usages for producing ECs by T7 RNAP.

Reagent usage (10 µM, 100 µL)
**Step 1** in the buffer (2 mM $MgSO_4$, 40 mM Tris-HCl, 100 mM K_2SO_4, 10 mM DTT, pH 8.0) at 37 ℃ for 15 min:<br>10 µM DNA-beads, 10 µM T7 RNAP, 1.12 mM ATP, 0.96 mM GTP and 32 µM UTP;<br><br>**Steps 2–15** in the buffer (2 mM $MgSO_4$, 40 mM Tris-HCl, pH 8.0) at 37 ℃ for 10 min:<br>**Step 2**: 40 µM ATP, 30 µM CTP, 10 µM UTP;<br>**Step 3**: 10 µM ATP, 10 µM CTP, 20 µM GTP;<br>**Step 4**: 20 µM ATP, 10 µM CTP, 40 µM UTP;<br>**Step 5**: 20 µM GTP, 10 µM UTP;<br>**Step 6**: 30 µM CTP, 10 µM Cy3-UTP;<br>**Step 7**: 50 µM CTP, 70 µM GTP, 30 µM UTP;<br>**Step 8**: 30 µM ATP, 10 µM GTP, 20 µM UTP;<br>**Step 9**: 10 µM GTP, 20 µM CTP, 10 µM UTP;<br>**Step 10**: 60 µM ATP, 40 µM GTP;<br>**Step 11**: 10 µM GTP, 10 µM Cy5-UTP;<br>**Step 12**: 30 µM ATP, 10 µM GTP, 20 µM UTP;<br>(the dissociated EC-81 used for smFRET)<br>**Step 13**: 30 µM ATP, 10 µM CTP, 10 µM UTP<br>(the dissociated EC-86 used for smFRET)<br><br>**Step 13 can be replaced by steps I, II, III and IV:**<br>**Step I**: 10 µM CTP (the dissociated EC-82 used for smFRET);<br>**Step II**: 10 µM ATP (the dissociated EC-83 used for smFRET);<br>**Step III**: 10 µM UTP (the dissociated EC-84 used for smFRET);<br>**Step IV**: 20 µM ATP;<br><br>**Step 14**: 30 µM ATP, 10 µM CTP, 10 µM GTP<br>(the dissociated EC-91 used for smFRET);<br>**Step 15**: 10 µM CTP, 20 µM GTP, 10 µM UTP.

Supplementary Table 5 | Reagent usages for testing processive activity of EC-59.

**Reagent usage**
**For preparing EC-59 (Steps 1 to 6):**<br>**Step 1** in the buffer (2 mM $MgSO_4$, 40 mM Tris-HCl, 100 mM K_2SO_4, 10 mM DTT, pH 8.0) at 37 ℃ for 15 min:<br>10 μM DNA-beads, 10 μM T7 RNAP, 1.12 mM ATP, 0.96 mM GTP and 32 μM UTP;<br><br>**Steps 2–6** in the buffer (2 mM $MgSO_4$, 40 mM Tris-HCl, 10 mM DTT, pH 8.0) at 37 ℃ for 10 min:<br>**Step 2:** 40 μM ATP, 30 μM CTP, 10 μM UTP;<br>**Step 3:** 10 μM ATP, 10 μM CTP, 20 μM GTP;<br>**Step 4:** 20 μM ATP, 10 μM CTP, 40 μM UTP;<br>**Step 5:** 90 μM GTP, 80 μM CTP, 50 μM UTP;<br>**Step 6:** 30 μM ATP, 10 μM GTP, 20 μM UTP;<br>**Collect the dissociated EC-59 separated from solid phase after SPE.**<br><br>**For detecting activity of EC-59:**<br>Add 7.5 μM ATP, 2.5 μM CTP, 5 μM GTP and 3 μM UTP to the dissociated EC-59, incubate at 37 ℃ for 10 min.

Supplementary Table 6 │ Reagent usages in 8 step-PLOR to generate 2AP-riboSAM and 2AP-mutant.

**Reagent usage (10 µM, 1 mL)**
**Step 1** in the buffer (6 mM $MgSO_4$, 40 mM Tris-HCl, 100 mM K_2SO_4, 10 mM DTT, pH 8.0) at 37 ℃ for 15 min:<br>10 µM DNA-beads, 10 µM T7 RNAP, 1.12 mM ATP, 0.96 mM GTP and 32 µM UTP;<br>**For 2AP-mutant:** 10 µM DNA-beads, 10 µM T7 RNAP, 0.96 mM ATP, 1.20 mM GTP and 64 µM UTP;<br><br>**Steps 2–8** in the buffer (6 mM $MgSO_4$, 40 mM Tris-HCl, 10 mM DTT, pH 8.0) at 25 ℃ for 10 min:<br>**Step 2**: 40 µM ATP, 30 µM CTP, 10 µM UTP;<br>**For 2AP-mutant:** 20 µM ATP, 40 µM CTP, 10 µM UTP;<br>**Step 3**: 10 µM ATP, 10 µM CTP, 20 µM GTP;<br>**Step 4**: 20 µM ATP, 10 µM CTP, 40 µM UTP;<br>**Step 5**: 90 µM GTP, 80 µM CTP, 50 µM UTP;<br>**Step 6**: 30 µM ATP, 10 µM UTP;<br>**Step 7**: 10 µM GTP, 10 µM 2AP-triphosphate (2AP-TP);<br>**Step 8**: 150 µM ATP, 50 µM CTP, 100 µM GTP, 60 µM UTP.

Supplementary Table 7 | Reagent usages in 12 step-PLOR to generate 37Cy3-74Cy5-riboSAM.

**Reagent usage (10 μM, 1 mL)**
**Step 1** in the buffer (6 mM $MgSO_4$, 40 mM Tris-HCl, 100 mM K_2SO_4, 10 mM DTT, pH 8.0) at 37 ℃ for 15 min:<br>10 μM DNA-beads, 10 μM T7 RNAP, 1.12 mM ATP, 0.96 mM GTP and 32 μM UTP;
**Steps 2–12** in the buffer (6 mM $MgSO_4$, 40 mM Tris-HCl, 10 mM DTT, pH 8.0) at 25 or 30 ℃ for 10 min:<br>**Step 2**: 40 μM ATP, 30 μM CTP, 10 μM UTP;<br>**Step 3**: 10 μM ATP, 10 μM CTP, 20 μM GTP;<br>**Step 4**: 20 μM ATP, 10 μM CTP, 40 μM UTP;<br>**Step 5**: 20 μM GTP, 10 μM UTP;<br>**Step 6**: 30 μM CTP, 10 μM Cy3-UTP (30 ℃, 10 min);<br>**Step 7**: 50 μM CTP, 70 μM GTP, 30 μM UTP;<br>**Step 8**: 30 μM ATP, 10 μM GTP, 20 μM UTP;<br>**Step 9**: 10 μM GTP, 20 μM CTP, 10 μM UTP;<br>**Step 10**: 60 μM ATP, 40 μM GTP;<br>**Step 11**: 10 μM GTP, 10 μM Cy5-UTP (30 ℃, 10 min);<br>**Step 12**: 90 μM ATP, 30 μM CTP, 40 μM GTP, 40 μM UTP.

Supplementary Table 8 ｜ Reagent usages in 8 step-PLOR to generate 16Cy3-62Cy5-riboSAM.

**Reagent usage (10 µM, 1 mL)**
**Step 1** in the buffer (6 mM $MgSO_4$, 40 mM Tris-HCl, 100 mM K_2SO_4, 10 mM DTT, pH 8.0) at 37 ℃ for 15 min:<br>10 µM DNA-beads, 10 µM T7 RNAP, 1.12 mM ATP, 0.96 mM GTP and 32 µM UTP;<br><br>**Steps 2–8** in the buffer (6 mM $MgSO_4$, 40 mM Tris-HCl, 10 mM DTT, pH 8.0) at 25 or 30 ℃ for 10 min:<br>**Step 2**: 40 µM ATP, 30 µM CTP, 10 µM Cy3-UTP (30 ℃, 10 min);<br>**Step 3**: 10 µM ATP, 10 µM CTP, 20 µM GTP;<br>**Step 4**: 20 µM ATP, 10 µM CTP, 40 µM UTP;<br>**Step 5**: 90 µM GTP, 80 µM CTP, 50 µM UTP;<br>**Step 6**: 30 µM ATP, 10 µM GTP, 20 µM UTP;<br>**Step 7**: 10 µM GTP, 20 µM CTP, 10 µM Cy5-UTP (30 ℃, 10 min);<br>**Step 8**: 150 µM ATP, 30 µM CTP, 90 µM GTP, 50 µM UTP.

Supplementary Table 9 | Reagent usages in 5 step-PLOR to generate ^{15}N-16U24G-riboSAM.

Reagent usage (20 µM, 40 mL)
**Step 1** in the buffer (6 mM $MgSO_4$, 40 mM Tris-HCl, 100 mM K_2SO_4, 10 mM DTT, pH 8.0) at 37 ℃ for 15 min:<br>20 µM DNA-beads, 20 µM T7 RNAP, 2.24 mM ATP, 1.92 mM GTP and 64 µM UTP;<br><br>**Steps 2–5** in the buffer (6 mM $MgSO_4$, 40 mM Tris-HCl, 10 mM DTT, pH 8.0) at 25 ℃ for 10 min:<br>**Step 2**: 80 µM ATP, 60 µM CTP, 20 µM ^{15}N-UTP;<br>**Step 3**: 20 µM ATP, 20 µM GTP;<br>**Step 4**: 20 µM CTP, 20 µM ^{15}N-GTP;<br>**Step 5**: 400 µM ATP, 280 µM CTP, 400 µM GTP, 340 µM UTP.

Supplementary Table 10 | Reagent usages in 7 step-PLOR to generate ^{15}N-25U32G-riboSAM.

Reagent usage (20 µM, 40 mL)
**Step 1** in the buffer (6 mM $MgSO_4$, 40 mM Tris-HCl, 100 mM K_2SO_4, 10 mM DTT, pH 8.0) at 37 ℃ for 15 min:<br>20 µM DNA-beads, 20 µM T7 RNAP, 2.24 mM ATP, 1.92 mM GTP and 64 µM UTP;
**Steps 2–7** in the buffer (6 mM $MgSO_4$, 40 mM Tris-HCl, 10 mM DTT, pH 8.0) at 25 ℃ for 10 min:<br>**Step 2**: 80 µM ATP, 60 µM CTP, 20 µM UTP;<br>**Step 3**: 20 µM ATP, 20 µM CTP, 40 µM GTP;<br>**Step 4**: 20 µM ^{15}N-UTP;<br>**Step 5**: 40 µM ATP, 20 µM CTP, 60 µM UTP;<br>**Step 6**: 20 µM ^{15}N-GTP;<br>**Step 7**: 360 µM ATP, 260 µM CTP, 380 µM GTP, 260 µM UTP.

Supplementary Table 11 │ Reagent usages in 10 step-PLOR to generate 16Cy3-74Cy5-riboSAM or 16Cy3-74Cy5-mutant.

**Reagent usage (10 μM, 1 mL)**
**Step 1** in the buffer (6 mM $MgSO_4$, 40 mM Tris-HCl, 100 mM K_2SO_4, 10 mM DTT, pH 8.0) at 37 ℃ for 15 min:<br>10 μM DNA-beads, 10 μM T7 RNAP, 1.12 mM ATP, 0.96 mM GTP and 32 μM UTP;<br>**For 16Cy3-74Cy5-mutant:** 10 μM DNA-beads, 10 μM T7 RNAP, 0.96 mM ATP, 1.20 mM GTP and 64 μM UTP;<br><br>**Steps 2–10** in the buffer (6 mM $MgSO_4$, 40 mM Tris-HCl, 10 mM DTT, pH 8.0) at 25 or 30 ℃ for 10 min:<br>**Step 2**: 40 μM ATP, 30 μM CTP, 10 μM Cy3-UTP (30 ℃, 10 min);<br>**For 16Cy3-74Cy5-mutant:** 20 μM ATP, 40 μM CTP, 10 μM Cy3-UTP (30 ℃, 10 min);<br>**Step 3**: 10 μM ATP, 10 μM CTP, 20 μM GTP;<br>**Step 4**: 20 μM ATP, 10 μM CTP, 40 μM UTP;<br>**Step 5**: 90 μM GTP, 80 μM CTP, 50 μM UTP;<br>**Step 6**: 30 μM ATP, 10 μM GTP, 20 μM UTP;<br>**Step 7**: 10 μM GTP, 10 μM UTP, 20 μM CTP;<br>**Step 8**: 60 μM ATP, 40 μM GTP;<br>**Step 9**: 10 μM GTP, 10 μM Cy5-UTP (30 ℃, 10 min);<br>**Step 10**: 90 μM ATP, 40 μM GTP, 30 μM CTP, 40 μM UTP.

Supplementary Table 12 | Peak intensities and K_d value measured from the NMR spectra of ^{15}N-25U32G-riboSAM at 25 ℃.

Peak	Peak intensity	Peak percentage (%)	K_d (μM)
a	2.0218 e+005	11.2	27.9
t-p	3.1848 e+005	17.6	
h-p	6.1927 e+005	34.3	
t	2.9923 e+005	16.6	
h	3.6588 e+005	20.3	

Peak percentages were obtained by dividing the peak intensity by the sum of peak intensities. K_d was calculated by using the equation: $K_d = C_{apo} \times C_{SAM}/(C_{holo}+C_{transit})$, where C_{apo}, C_{SAM}, C_{holo} and $C_{transit}$ are the concentrations of *apo*, SAM, *holo* and *transit*. And C_{apo}, C_{holo} and $C_{transit}$ were calculated by using the equation: C =0.15 mM × peak percentage. And C_{SAM} = 0.15 mM- C_{holo}- $C_{transit}$.

Supplementary Table 13 | FPS-estimated results for *apo*, *transit* and *holo* structures of riboSAM.

**State**	**R_{mp} (Å)**	**$<R_{DA}>_E$ (Å)**	**sigmaDA (Å)**	**E_{FRET}**
apo	57.3	57.7	16.9	0.349
holo	32.0	44.6	13.8	0.716
transit	18.2	37.6	12.8	0.869

R_{mp} is the mean position distance. $<R_{DA}>_E$ represents the averaged distance between the FRET pair, and sigmaDA is its standard deviation. E_{FRET} is the FRET efficiency. R_0 of 52 Å was used in the calculation. The parameters used for Cy3 include W_{link} of 4.5 Å, $R_{dye(1)}$ 6.8 Å, $R_{dye(2)}$ 3.0 Å, $R_{dye(3)}$ 1.5 Å, linker of 30 Å. The parameters used for Cy5 include W_{link} of 4.5 Å, $R_{dye(1)}$ 11.0 Å, $R_{dye(2)}$ 3.0 Å, $R_{dye(3)}$ 1.5 Å, linker of 30 Å.

Supplementary Table 14 │ The DNA/RNA sequences used for producing ECs of riboSAM by *E. coli* RNAP.

DNA/RNA	Sequence
37Cy3-63 nt-RNA	5′-GGGAAGAAAUAACAAUCAACGACGUAUCAUUGUGCC U(Cy3)CGCUUUCCGCGGGGCGAUAAGUCCUG
DNA template	5′-GAATGGAGTACCGATGTTCTTATGTCATATCATCCCTTTC TTCAGGACTTATCATGGCTGTAAGTATCC-Desthiobiotin
DNA non-template	5′-GGATACTTACAGCCATTCGCCTAAACATAGAAAGGGATG ATATGACATAAGAACATCGGTACTCCATTC

Supplementary Table 15 │Reagent usages for producing ECs of riboSAM by *E. coli* RNAP.

**Reagent usage (5 µM, 100 µL)**
**EC assembly** in the buffer (2 mM $MgSO_4$, 40 mM Tris-HCl, 10 mM DTT, pH 8.0) at 37 ℃ for 90 min:<br>5 µM DNA-beads, 5 µM 37Cy3-63nt-riboSAM and 5 µM *E. coli* RNAP.<br><br>**Steps 1**–**3** in the buffer (2 mM $MgSO_4$, 40 mM Tris-HCl, pH 8.0) at 37 ℃ for 10 min:<br>**Step 1**: 30 µM ATP, 20 µM GTP;<br>**Step 2**: 5 µM GTP, 5 µM azide-UTP;<br>**Step 3**: 15 µM ATP, 5 µM GTP, 10 µM UTP;<br><br>**Step 4** in the buffer (2 mM $MgSO_4$, 40 mM Tris-HCl, pH 8.0) at 37 ℃ for 60 min: 0.5 mM DBCO-Cy5 (the dissociated EC-81 used for smFRET);<br><br>**Step 5**: 15 µM ATP, 5 µM CTP, 5 µM UTP<br>(the dissociated EC-86 used for smFRET)<br>**Step 6**: 15 µM ATP, 5 µM CTP, 5 µM GTP (the dissociated EC-91 used for smFRET).

Supplementary Table 16 │ K_{obs} values for 2AP-riboSAM or its mutant measured by stopped-flow fluorescence experiments at 25 ℃.

RNA	SAM concentration (μM)	Average K_{obs} (s^{-1})
2AP-riboSAM (0.5 μM)	1	0.097 ± 0.0005
	2	0.107 ± 0.0032
	3	0.114 ± 0.0028
	4	0.121 ± 0.0018
	5	0.127 ± 0.0036
	10	0.149 ± 0.0011
	25	0.227 ± 0.0034
	50	0.377 ± 0.0020
2AP-mutant (0.5 μM)	1	0.108 ± 0.0007
	2	0.110 ± 0.0011
	3	0.114 ± 0.0025
	4	0.121 ± 0.0009
	5	0.128 ± 0.0009
	10	0.143 ± 0.0049
	25	0.202 ± 0.0005
	50	0.325 ± 0.0042
